# Adermatoglyphia: Barriers to Biometric Identification and the Need for a Standardized Alternative

**DOI:** 10.7759/cureus.4040

**Published:** 2019-02-08

**Authors:** Nuraiz Sarfraz

**Affiliations:** 1 Internal Medicine, King Edward Medical University/Mayo Hospital, Faisalabad, PAK

**Keywords:** finger printing, adermatoglyphia, loss of fingerprints, biometrics, identification and authentication

## Abstract

Arguably, fingerprinting is the single most widely utilized method for individual identification and authentication (I&A). Dermatoglyphics form a vital portion of mass data collection, biometric scrutiny, and verification. Adermatoglyphia, or simply, loss of fingerprints attributed to a medical cause, represents a taxing situation for such biometric scrutiny systems requiring a fingerprint scan as a mandatory phase in I&A procedure. The scenario can be extremely debilitating for the adermatoglyphia patients, especially when the condition is permanent or irreversible. This article reviews different causes of adermatoglyphia, the challenge it poses to biometric identification, and the potential substitute modalities for fingerprinting technology. These modalities can function as a backup program for biometric surveillance in both medical and non-medical settings under circumstances when the fingerprinting method fails to comply.

## Introduction and background

Dermatoglyphics (from the roots “derma” for skin and “glyphos” for carvings) is the study of various integumentary ridge patterns that form on fingertips, toes, palms of the hands, and soles of the feet. Fingerprinting describes the technical aspects of recording the skin ridge pattern present on fingertips, establishing one of the key components of human identification. More than a century ago, Sir Francis Galton discovered that these ridge patterns are astoundingly constant throughout the lifespan of an individual [1]. Later, the term “dermatoglyphics” was introduced by Dr. Harold Cummins in 1936 [2].

Fingerprinting is the most commonly utilized method for human identification and authentication (I&A) [3]. It is an integral component of a personal profile and biodata. Most government and private sectors seeking or involving personal biometric information require individuals to be fingerprinted to complete routine biometric record and documentation. Not only does fingerprinting concern the conventional domains of clinical medicine, forensic science, anthropometry, criminology, customs, and security agencies [3], its applicability has been incorporated into every so-called formal “security screen”. The spectrum of its utility ranges from gaining access to sensitive computerized documents and handling transactions to checking-in for daily office work and unlocking mobile phones and electronic devices [4-5].

**Figure 1:**
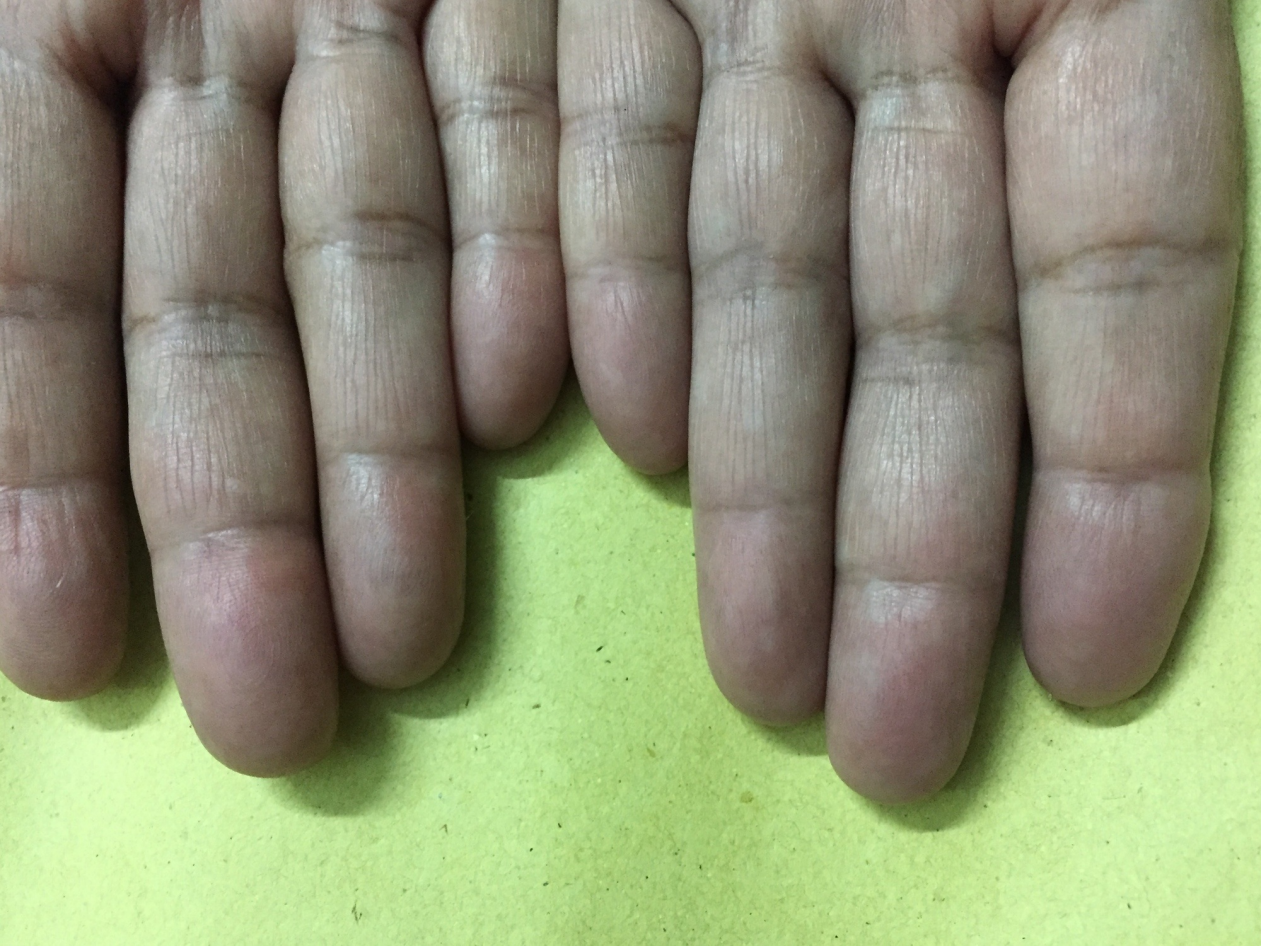
Acquired idiopathic adermatoglyphia in a 59-year-old female patient showing fingers of both hands This woman visited the dermatology department in July 2016 for medical evaluation. She realized the loss of her fingerprints when she was unable to renew her national identity card, requiring her to be ten-printed (i.e., all fingers and thumbs of both hands were scanned/printed). Except for hypertension well-controlled with medication, her past medical history, physical exam, and laboratory reports failed to identify any probable cause of her findings. A detailed physical exam revealed marked effacement of ridges, involving fingers of both hands (Image courtesy of Dermatology Department, Mayo hospital, Lahore).

**Figure 2:**
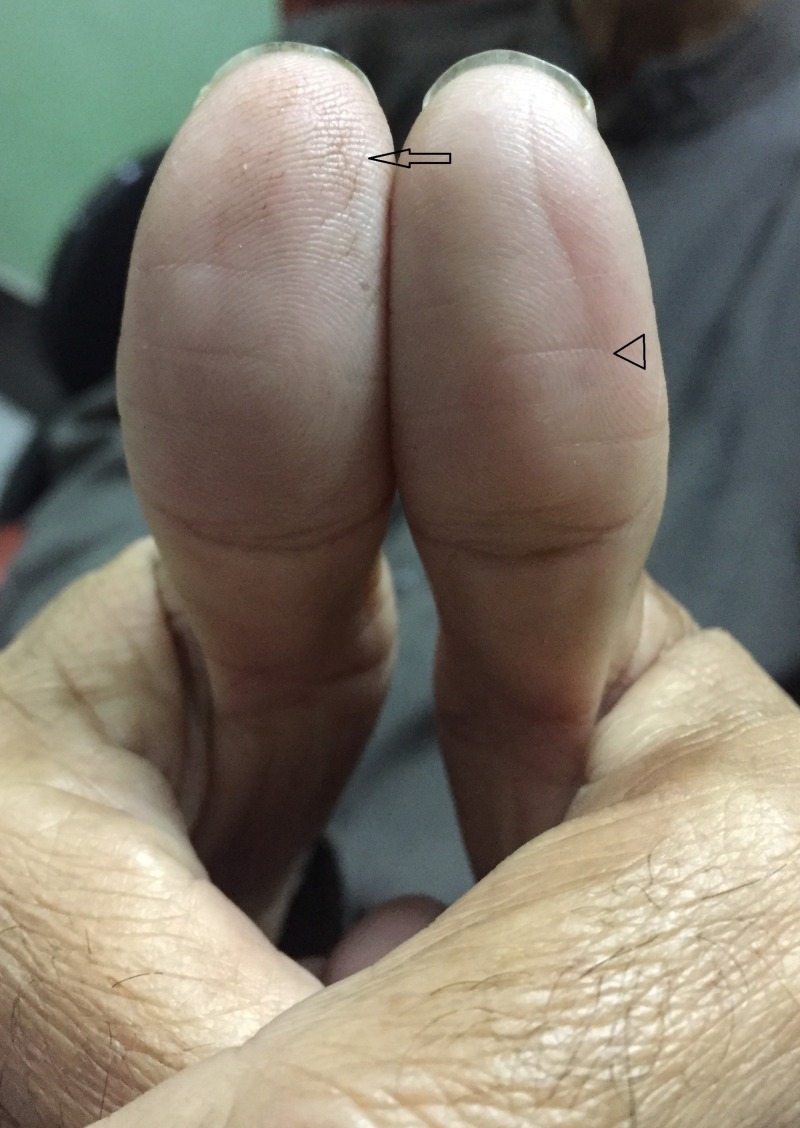
Geriatric atrophy of epidermal ridges in a 66-year-old male patient showing thumbs of both hands This patient presented in November 2017 to obtain a medical certificate to access his bank account. The bank accounts were being biometrically verified, and repeated attempts to scan his fingertips failed to record any discernible pattern. Note the atrophy of epidermal ridges around the center of digital pulp with fissuring “black arrow” along with the formation of transverse creases “arrowhead” (Image courtesy of Dermatology Department, Mayo hospital, Lahore).

Adermatoglyphia is clinically defined as the congenital or an acquired loss of this epidermal ridge pattern [6]. It can be limited to a few digits or all fingers and can also refer to the absence of the ridge patterns formed on the plantar aspects of the feet. Furthermore, adermatoglyphia can refer to a partial loss of the ridges (i.e., ridges are unnoticeable on general evaluation but detectable on deep inspection or under a magnifying lamp) or a complete absence (depicting total effacement) of epidermal ridges. Such scenarios present time-consuming challenges not only for the authorities but also for the concerned individual, in completing verification procedures (Figures 1, 2). Face recognition and fingerprinting are the primary modes of biometric I&A worldwide. Any technical problem or error hampering this series of verification steps can cause the entire verification process to come to a halt [7]. This article discusses the obstacles faced by patients suffering from adermatoglyphia undergoing a mandatory biometric fingerprinting procedure. Additionally, this review highlights the necessity of a standardized, globally acceptable criterion that can act as a biometric substitute protocol. Such a biometric substitute system can function as a backup option under circumstances when the conventional fingerprinting verification fails or is not applicable (e.g., patients suffering from adermatoglyphia or amputated limbs).

## Review

Fingerprints are important dermatological landmarks with substantial applications in medicine, forensics, anthropology, and security. The epidermal ridges usually develop around 10 to 17 weeks post-fertilization, and their formation is influenced by genetic and environmental components [6]. The development affected by multiple genes during embryogenesis results in the formation of a perpetually unique pattern persisting throughout the lifespan of an individual [6]. However, there are certain congenital and acquired conditions that can affect the integrity of dermatoglyphics (Tables 1-3).

**Table 1:** Genetic disorders with abnormal dermatoglyphics AD, autosomal dominant; ATD angle, axial tri-radii angle [3]

Diseases	Genetics	Dermatoglyphic Features
Down syndrome	Trisomy 21	Simian line, fewer ridges along digital midline, distinct ATD angle, predominance of ulnar loops
Turner syndrome	45, X0	Predominance of whorls depending on the type of chromosomal abnormality
Klinefelter syndrome	47, XXY	Excessive arches on digit 1, more ulnar loops on digit 2, fewer whorls, lower finger ridges
Edwards’ syndrome	Trisomy 18	6 to 10 arches on fingertips and Simian crease in 30% of cases
Patau syndrome	Trisomy 13	Excess arches on fingertips, polydactyly and Simian line in 60% cases
Noonan syndrome	Multiple genes/AD	Increased whorls on fingertips and axial tri-radius, Simian line in certain cases

**Table 2:** Congenitally abnormal dermatoglyphics with relative physical and clinical associations AD, autosomal dominant inheritance; NA, not applicable [3,6-9]

Categories	Trait	Features	Associations
Ridge aplasia	AD	Absence over entire palmoplantar surfaces; muted SMARCAD1 helicase isoforms in certain cases	Congenital facial milia, acral blistering, digital contractures and nail abnormalities in rare Basan Syndrome [8]
Ridge hypoplasia	AD	Not absents but less conspicuous	Excess of white lines on the prints
Ridge dissociation	AD/sporadic	Ridge dots, enclosures, bifurcations and crosses, may be mistaken as scarring	Found in some patients with schizophrenia, Down syndrome, epilepsy, and albinism.
Ridges-off-the-end	AD	Instead of running transversely, ridges run vertically off the fingertips	NA
Ridges-off-the-end with dissociation	NA	A combination of ridge dissociation and ridges running vertically off the end [9].	NA

**Table 3:** Transient and permanent causes of acquired adermatoglyphia GVHD, graft versus host disease; SSS, scalded skin syndrome; LE, lupus erythematosus [3,7,10-11]

Dermatological Causes	Non-dermatological Causes
Dermatitis	Accidental
1. Contact dermatitis	1. Trauma
2. Allergic dermatitis	2. Burns
3. Atopic eczema	3. Amputation
4. Dyshidrotic eczema	4. Caustic abrasion
5. Miscellaneous dermatitis affecting volar surfaces	5. Denervation injury
Infectious	Drug-related
1. Scabies	1. Potent topical steroids
2. Herpetic Whitlow	2. Capecitabine chemotherapy
3. Pyoderma/Impetigo		
4. Pitted keratolysis	Medical disorders
5. Palmar warts		
6. Lepromatous leprosy	1. Metabolic/medical disorders causing neuropathy
7. Syphilitic rash	2. Celiac disease
8. Coxsackievirus A rash	3. Malnutrition/Nutritional deficiencies
9. Scarlet fever/SSS		
	Miscellaneous	
Drug-induced Rash	1. Occupational micro-abrasions
	2. Prolonged liquid immersion	
1. Erythema multiforme	3. Idiopathic
2. Steven Johnson syndrome		
3. Toxic epidermal necrolysis	4. Iatrogenic/Dermatitis artefacta
4. Serum sickness/GVHD	5. Normal aging
Immune-mediated	
1. Cutaneous LE		
2. Epidermolysis bullosa		
3. Pemphigus vulgaris		
4. Systemic sclerosis		
5. Psoriasis		
6. Kawasaki’s disease		
7. Dermatitis herpetiformis		
8. Keratoderma blennorrhagica		
Miscellaneous		
1. Gangrene		
2. Lichen		
3. Primary Hyperhidrosis		
4. Keratoderma		
5. Xanthoma striatum palmare		
6. Acanthosis nigricans		
7. Acrodermatitis enteropathica		
8. Dermatoses involving volar surface of fingers		

Epidemiology of adermatoglyphia

An extensive literature review to evaluate the prevalence of fingerprint loss among the general population due to congenital and acquired disease yielded a paucity of data. In a national survey conducted in a Lebanese population in 2013, the incidence of absent fingerprints was 0.18%, predominantly affecting those in the geriatric age group (aged 65 years or older) and women more than men [7]. The incidence of loss of fingerprints was 0.3% for those 24 years or younger, 2.8% for those aged 25 to 64 years, and 8.5% for those aged 65 years or older. For cases where absent fingerprints were attributed to dermatological causes, the number of female patients was 3.75 times greater than the number of male patients [7]. Most of these female patients were housewives.

Congenital causes of adermatoglyphia

Some genetic disorders can indirectly predispose a person to abnormal fingerprint configurations (Table 1 illustrates a few such causes) [3]. Furthermore, conditions that directly affect the gross morphology of the fingers (e.g., polydactyly, syndactyly, brachydactyly, and ectrodactyly) can also skew the conventional parameters of biometric fingerprinting procedure [7]. Congenitally abnormal dermatoglyphics can be categorized into five basic domains (Table 2). Such phenotypic variants are rarely encountered in the general population [3,6-9].

Acquired causes of adermatoglyphia

Acquired causes substantially eclipse the congenital causes of abnormal dermatoglyphics. Indeed, the loss of epidermal ridges is not an uncommon observation, especially in the elderly population (i.e., those aged 65 years or above). Interestingly, one study found adermatoglyphia to be more common in women than men [7]. Acquired adermatoglyphia can be further classified by dermatological and non-dermatological causes (Table 3). Trauma, amputation, and burns are among the more commonly encountered, accidental, non-dermatological etiologies. In the Lebanese national survey by Haber et al., dermatological causes accounted for 52.9% of all cases of adermatoglyphia, with dermatitis contributing to the majority of these cases (61%) [7].

Certain drugs like potent corticosteroids can cause epidermal ridge atrophy [10], and in recent years, capecitabine-induced adermatoglyphia has resulted in immigration delays at airports and travel warnings for patients undergoing capecitabine chemotherapy [11]. The list of acquired causes of adermatoglyphia is quite extensive (Table 3), highlighting the gravity of this issue in the general population [3,7,10-11].

Methods of human identification and authentication

An in-depth analysis of medical literature was conducted involving MEDLINE and PubMed medical databases, including ResearchGate and using the following terms: adermatoglyphia, dermatoglyphics, loss of fingerprints, and biometrics both individually and in combination. Surprisingly, no articles were found in the scientific literature discussing specific guidelines or a protocol for adermatoglyphia patients undergoing a mandatory fingerprint verification. The main objective of this article was to signify the necessity of a second-line substitute I&A system, especially for patients suffering from irreversible adermatoglyphia. Issuing a medical certificate is medico-legally imperative but only provides a temporary solution, as the validity and the authenticity of such certificates might not be acceptable by other organizations.

Furthermore, different set-ups demand different types of biometric verification, often involving mandatory, multi-modal biometric functions [12-13] operating without a backup plan. Additionally, some corporations require only thumb-printing for monitoring office attendances or bank account verification. Other agencies granting citizenship, driver’s licenses, passports, and immigration papers require a more stringent policy, requiring individuals to be ten-printed. Therefore, a substitute I&A system is recommended that can function globally as a default program for patients suffering from irreversible adermatoglyphia.

Biometrics

Over the past few decades, many advancements have been made in biometric technology with the introduction of new measurable biological traits as potential biometric targets. Table 4 presents a concise review of the indispensable characteristics required for a biometric indicator. While many of these biological traits fulfill the necessary characteristics (Table 4) required to qualify as a biometric indicator, feasibility becomes the most important factor based upon efficacy, social acceptability, the technical complexity of systems, ease of applicability, and cost-effectiveness. Consequently, there is no single biometric indicator that ideally achieves all these parameters. Therefore, hybrid or complex multi-modal biometric systems are used to enhance the accuracy and efficacy of I&A [12-13].

**Table 4:** Essential qualities of a biometric indicator

Characteristic	Definition
Generality	Universally present in all individuals
Uniqueness	No individuals share the same configuration
Stability	Unchanging throughout the lifespan
Quantifiable	Measurable for comparison

**Figure 3:**
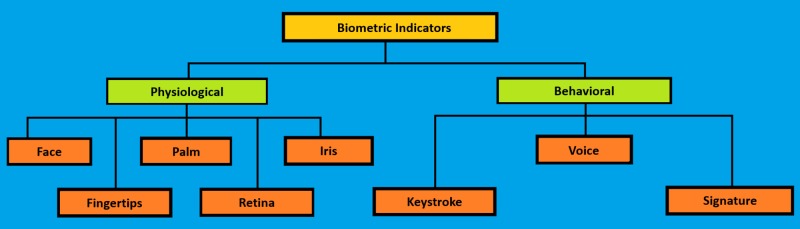
Biometric sub-types Classification is based on physiological and behavioral traits [14-15]

Biometric traits can be divided into physiological subtypes (e.g., fingerprinting, face recognition, hand geometry, and iris and retinal scan), and behavioral subtypes (e.g., signatures, keystroke, and voice and gait patterns; Figure 3) [14-16].

In addition to the facial recognition and fingerprinting technology, extensive research has been conducted on other biometric recognition techniques, namely, iris/retinal scanning, hand geometry and palm prints, DNA fingerprinting, voice recognition, signature scanning, gait patterns, and keystroke patterns [14-17]. Future endeavors will involve the exploration of other anatomical organs and parts, with the potential for I&A to fulfill essential biometric features (Table 4). These domains include, but are not limited to, finger-vein patterns [15], foot-printing [18], dorsal finger pattern [19], lip-printing [20], and tongue-printing [21].

Electrocardiography (ECG) has also been evaluated as a tool for biometric identification in recent years [22] using peculiar wave-forms (i.e., the peaks and intervals formed by P, Q, R, S, and T waves). Based upon the specific temporal and amplitude sequences recorded on these points, a set of signals are generated to form a feature extraction that can be deployed to discern one individual from another [23]. Compared to other modalities, the main advantage of this method lies in the impossibility to self-mutilate one’s own cardiac activity. However, the cardiac activity does not remain constant over a person’s lifetime due to a pathological process or aging. Consequently, biometric ECG can identify individuals but has limited capacity to verify identity over longer periods.

Radio-frequency Identification Technology

Radio-frequency identification (RFID) uses radio signals as a medium to transfer I&A data [24]. A small RFID device, usually a microchip, is incorporated into the desired object and emits radio waves detected by an RFID reader. The object can be a product, animal, or a human being, and given the diverse spectrum of its applicability, RFID technology has already penetrated multiple sectors of healthcare, security agencies, agriculture, and manufacturing industries [24]. These RFID tags accommodate data for purposes that range between storing an identification number to incorporating complex details about a product or a person.

Electronic passports are an extension of RFID technology and contain stored personal biometrics [25]. This allows for border and custom securities at airports to conduct I&A instantly without scanning an individual's face or fingerprints [26]. The RFID chip is incorporated into the main data-page, providing 16 distinct data groups. The first five data groups essentially store critical information related to I&A. Table 5 presents components of an RFID chip inserted in an e-passport. Note that data groups 2-4 specifically stores the biometric data of that individual [26].

**Table 5:** Data groups of an e-passport DG, data group [26]

Data Group	Details of Stored Information
DG1	Document Details
DG2	Encoded face
DG3	Encoded fingerprints
DG4	Encoded iris
DG5	Displayed portrait
DG6	Reserved for future use
DG7	Displayed signatures or usual mark
DG8, DG9, & DG10	Data features
DG11, DG12, & DG13	Additional personal and document details
DG14	Reserved for future use
DG15	Active authentication public key
DG16	Person(s) to notify
SDE	Security data elements

Another similar application is the insertion of an RFID implant into a person’s body. These microchip implants are also instrumental in medical settings, especially for patients suffering from a life-threatening medical condition, dementia, communicative disorder or for patients with debilitating psychiatric diseases [27]. Unlike RFID-integrated documents (e.g., e-passport or a smartcard), RFID implants cannot be lost or forgotten. However, implants carry certain privacy and security risks because malicious agencies using compatible RFID readers could access, block or even alter sensitive biometric data stored in the implant [27]. Consequently, the integration of cryptographic features can resolve this issue to some extent by providing encryption on the database. Nevertheless, the RFID implant technology use raises concerns that have yet to be resolved, related to intrinsic technical issues, privacy, ethical concerns, and health-related problems [27-29]. In the field of biometrics, RFID technology is still evolving and will likely offer practical solutions in the future.

## Conclusions

Adermatoglyphia, especially due to acquired causes, is more common than anticipated and is not an uncommon finding in the geriatric population and manual laborers. Despite its utterly benign nature, the relentless invasion of biometric I&A into modern life via fingerprint verification creates enormous hurdles for patients with irreversible adermatoglyphia. While advancements in biometric technology have rendered the process more rigorous and quicker, these systems are frequently deployed in various institutions and departments without a substitute or default alternative option that could function as a solution for patients with absent fingerprints. Therefore, specific guidelines or a substitute I&A system is recommended that can function globally, as a default program, facilitating I&A for patients suffering from irreversible adermatoglyphia.
